# Dr. J. S. Billings

**Published:** 1895-10-19

**Authors:** 


### Dr. John S. Billings.

On October 1st Lieutenant-Colonel and Deputy-Surgeon-General Billings voluntarily retired from the United States Army, after thirty-four years' service. There is no member of the medical profession in the English-speaking world who is more widely known, admired, and honoured by his professional brethren and by scientists generally than Dr. John S. Billings. He holds the degrees of five Universities, including those of Edinburgh, Harvard, Munich, and Oxford. His life's work has been the establishment and completion of the Army Medical Museum and Library at Washington, and the preparation and publication of the Index Catalogue of the Library of the Surgeon-General's Office, United States Army, which are monuments to his energy, capacity, and devotion. All who have seen that museum must recognise that it is on the whole probably the most complete and the best arranged in the world. No one who has visited it can have failed to have been impressed with a desire to master the system upon which it has been built up, the aims of its founder, and the comprehensive character of the plan upon which it has been developed. The Index Catalogue has now been completed, although the final volume has not yet been issued from the press. It represents an amount of learning, knowledge, capacity, and grit on the part of its author which has deservedly won for him a first place amongst men of letters all the world over. The index catalogue contains a bibliography of authors and subjects so complete and comprehensive that anyone engaged in research must perforce refer to its pages, because he will there find precise and full information which must spare him an immensity of labour, and render it possible for researches to be conducted which, previous to the publication of this gigantic work, were almost, if not quite, impossible.

Dr. Billings has been a prolific writer upon many subjects, including medical literature, hospitals, vital statistics, hygiene, and kindred topics. The addresses he has delivered at the International Medical Congresses and elsewhere have won for him wide and deserved fame. As the designer of the John Hopkins Hospital, Baltimore—probably the finest hospital in the world—he has shown how a hospital building can be made perfect in every department, and how it can be organised so as to yield the maximum of good to the patients, to science, and the world of medicine. For more than half a lifetime Dr. Billings has been identified with Washington life, and, in conjunction with his wife, a lady of special charm and social distinction, has done infinite good in a multitude of ways in the capital city of the United States. It must be a satisfaction to Dr. Billings to know that in every part of the world where great minds are engaged in scientific research there he is honoured and respected to an extent which is almost, if not quite, unique.

On Monday next Dr. Billings will assume the chair of hygiene and the directorship of the Laboratory of Hygiene of the University of Pennsylvania. It is scarcely twenty years since the first separate institution of this kind was established for hygiene, and at the present time there are not more than a dozen such laboratories specially built and fitted for that purpose in existence throughout the world. Of these the best known is probably that of the University of Munich, under the direction of Professor Pettenkofer, while the largest is that of Berlin. This Laboratory of Hygiene at Philadelphia has been specially designed by Dr. Billings, and bears the marks of the thoroughness with which he carries out every project to which he sets his hand. He has not only provided most complete and modern apparatus for the study of bacteria and microzoa, and of their development, products, and effects, but also the means for chemical investigations of air, water, food, sewage, secretions and excretions, and the products of bacterial growth; the examination of drinking waters and the effects of various impurities in waters; the effect of working in compressed air; the air of school and other rooms; the dust and germs of the air, its changes by respiration and by ventilation; the adulterations, preparation, and preservation of foods and drinks; the investigation of various soils; the effects of clothing, the various kinds of lighting, heating, &c., and methods of disinfection under varying conditions. In addition to this the drainage of the laboratory is on a double system, and is so arranged as to permit of the trial of new forms of traps, sinks, closets, &c. Such are a few of the many important subjects which will be studied and elaborated under Dr. Billings' direction at the new Laboratory of Philadelphia. Dr. Billings has already done magnificent work, but the new field of research upon which he is about to enter must afford him opportunities for valuable service to science of which he is certain to take the fullest advantage. All honour to Dr. Billings and to the great nation of which he is so distinguished a member. In offering him our congratulations upon the completion of the Index Catalogue, we welcome him with heartfelt cordiality at the commencement of his new labours, which cannot fail to be attended by important results for the well-being and welfare of the world.

